# An epidemiological study of cancer in adult twins born in Norway 1905–1945

**DOI:** 10.1054/bjoc.2001.1827

**Published:** 2001-06

**Authors:** T Iversen, S Tretli, E Kringlen

**Affiliations:** The Department of Oncology, Ullevaal Hospital, Oslo, Norway; The Cancer Registry of Norway, Oslo; Department of Psychiatry, University of Oslo, Norway

**Keywords:** twin, cancer, incidence

## Abstract

We have identified 23 334 individuals (40%) of twins born in Norway 1905–45 where both twins were alive in 1960 without malignant disease. These were linked to the Cancer Registry of Norway. A reduced risk of malignant disease was demonstrated among twins for all tumour sites combined; standardized incidence rate (SIR): 0.90 (95% CI 0.85–0.94) in females and 0.95 (95% Cl 0.90–0.99) in males. In both sexes, we observed a significant reduced incidence of malignant melanomas of the skin. The incidence of colorectal cancer tended to be reduced for both sexes. In females, the incidence of tumours of the central nervous system and lungs were reduced. We consider our findings are real, but cannot explain them. © 2001 Cancer Research Campaign http://www.bjcancer.com


British Journal of Cancer (2001) 84(11), 1463–1465
© 2001 Cancer Research Campaign
doi: 10.1054/ bjoc.2001.1827, available online at http://www.idealibrary.com on IDEAL®

http://www.bjcancer.com

### An epidemiological study of cancer in adult twins born in Norway 1905–1945

**T Iversen[1], S Tretli[2] and E Kringlen[3]**

[1]The Department of Oncology, Ullevaal Hospital, Oslo, Norway; [2]The Cancer Registry of Norway, Oslo; [3]Department of Psychiatry, University of Oslo, Norway

**Summary** We have identified 23 334 individuals (40%) of twins born in Norway 1905–45 where both twins were alive in 1960 without malignant disease. These were linked to the Cancer Registry of Norway. A reduced risk of malignant disease was demonstrated among twins for all tumour sites combined; standardized incidence rate (SIR): 0.90 (95% CI 0.85–0.94) in females and 0.95 (95% CI 0.90–0.99) in males. In both sexes, we observed a significant reduced incidence of malignant melanomas of the skin. The incidence of colorectal cancer tended to be reduced for both sexes. In females, the incidence of tumours of the central nervous system and lungs were reduced. We consider our findings are real, but cannot explain them. © 2001 Cancer Research Campaign http://www.bjcancer.com

**Keywords**: twin; cancer; incidence

Examination of periods at risk in human life in relation to later development of malignant disease has recently extended towards the intrauterine life (Trichopoulos and Lipworth, 1995). However, the study of the fetal period in people born a long time ago represents a problem. One way to obtain such information is to examine birth weight, and several studies have commented upon birth weight in relation to malignant disease in adult life (Tibblin et al, 1995; Michels et al, 1996; Platz et al, 1998; Ekbom et al, 2000, Stavola et al, 2000). However, birth weight may also be rather difficult to obtain of children born early in the 20th century, as the birth records very often are lost.

Twins are often born at an earlier gestational age than singletons and often with a reduced birth weight. A careful study of twins in Norway in the period 1919–1930 found that pregnancy in twins was 16 days shorter (mean) compared with singletons (Waaler, 1934).

Since 1967 all the births in Norway are registered in the Medical Birth Registry of Norway. However, the population in this registry has not yet reached main age groups for cancer except for malignant disease among children. Thus, this particular registry is for the time being of minor importance for examining the relationship between the intrauterine period and later malignant disease. To study such questions we initiated a large-scale project to identify twins born in Norway in the period 1905–1945 (The National Twin Register) and we have taken the opportunity to match this register against the National Cancer Registry. This has enabled us to examine cancer risk among twins compared with the general population.

### MATERIALS AND METHODS

In Norway, local clergymen were for a long time entrusted with registering all births for Statistics Norway. Until 1916, the returns to this register were based on the records in the parish registers. Since then, details of all infants have been entered in the birth register, which is a civil affair regardless of whether or not the parents belong to the state church. The twin register was established covering the period 1895–1945 (Kringlen, 1978). In Norway the 11-digit personal identity number for every individual in Norway has been in use since the autumn of 1964, was initially based on the census of 1960. Thus, we can identify only those twins who were alive in the period covered by the identity number. As the authorities did not allow us to contact the twins or their families, we could not study the zygosity of the twins.

Among twins born 1905–1945 we were able to identify 23 334 individuals with personal identity numbers in which both members of the twin pair were alive at the census of 1960 (representing 40% of the twins born in Norway in the period 1905–45). However, those who died before 1960 were lost and also some cases in which the female twin changed her family name on marriage. As our material covers twins born during 1905–1945 who were alive in 1960 with malignant disease diagnosed after 1960, we have lost information on childhood cancer occurring in children as our youngest twin was 15 years old in 1960. Twins are evenly distributed through the whole period. For these 23 334, the Cancer Registry of Norway was used to identify cancer cases among the twins.

Since 1952, physicians in Norway have been obliged by law to report all malignant lesions to the Cancer Registry. The Register has been evaluated and found to be practically complete for all solid tumours. A comprehensive evaluation of the Cancer Registry on 1 October 1970 showed that only 0.9% were not reported. A pilot study covering 2 counties for 1976 was undertaken in 1979 and found 100% completeness for the first county, but only 88% for the other county. However, another evaluation of this particular county was for the years 1960, 1975 and 1981 and indicated 100% coverage (Lund, 1981; the Cancer Registry, 1982; Harvei et al, 1996). The identity number for every individual living in Norway, combined with routine reports from all departments of pathology and clinical departments when a malignant disease is

*Received 3 November 2000*
*Revised 2 February 2001*
*Accepted 2 February 2001*

*Correspondence to:* T Iversen

**Table 1** Standardized incidence rate (SIR) and 95% confidence interval (CI) among 23 334 twins in Norway born 1905–1945 where both of the twins were identified. (Person years at risk: total 709 009, males 369 418 and females 339 591)

| Cancer site | ICD-7 code | Observed | Expected | SIR | CI |
|---|---|---|---|---|---|
| Lip | 140 | | | | |
| males | | 29 | 22.7 | 1.28 | 0.86–1.84 |
| females | | 0 | 2.7 | 0.0 | 0.00–1.36 |
| Oesophagus | 150 | | | | |
| males | | 19 | 22.2 | 0.85 | 0.45–1.74 |
| females | | 5 | 6.3 | 0.79 | 0.26–1.85 |
| Stomach | 151 | | | | |
| males | | 136 | 129.4 | 1.05 | 0.89–1.24 |
| females | | 71 | 70.4 | 1.01 | 0.79–1.27 |
| Colon | 153 | | | | |
| males | | 128 | 155.43 | 0.82 | 0.69–0.98 |
| females | | 146 | 160.4 | 0.91 | 0.77–1.07 |
| Rectum | 154 | | | | |
| males | | 85 | 102.9 | 0.83 | 0.66–1.02 |
| females | | 58 | 75.7 | 0.77 | 0.58–0.99 |
| Pancreas | 157 | | | | |
| males | | 67 | 63.3 | 1.06 | 0.82–1.34 |
| females | | 37 | 48.5 | 0.76 | 0.54–1.05 |
| Larynx | 161 | | | | |
| males | | 28 | 26.2 | 1.07 | 0.71–1.54 |
| females | | 2 | 2.6 | 0.77 | 0.09–2.77 |
| Trachea, bronchus and lung | 162 | | | | |
| males | | 249 | 263.0 | 0.95 | 0.84–1.07 |
| females | | 60 | 79.8 | 0.75 | 0.57–0.97 |
| Breast | 170 | | | | |
| males | | 3 | 2.7 | 1.13 | 0.23–3.30 |
| females | | 376 | 403.0 | 0.93 | 0.84–1.03 |
| Cervix uteri | 171 | 99 | 104.4 | 0.95 | 0.77–1.15 |
| Corpus uteri | 172 | 100 | 101.1 | 0.99 | 0.81–1.20 |
| Ovary | 175 | 133 | 109.9 | 1.21 | 1.02–1.43 |
| Vulva/Vagina | 176 | 22 | 17.5 | 1.26 | 0.79–1.90 |
| Prostate gland | 177 | 358 | 339.8 | 1.05 | 0.95–1.17 |
| Testis | 178 | 27 | 22.6 | 1.20 | 0.79–1.74 |
| Kidney | 180 | | | | |
| males | | 57 | 72.0 | 0.79 | 0.60–1.03 |
| females | | 28 | 39.9 | 0.70 | 0.47–1.02 |
| Bladder | 181 | | | | |
| males | | 135 | 140.4 | 0.96 | 0.81–1.14 |
| females | | 32 | 39.8 | 0.80 | 0.55–1.13 |
| Melanoma of the skin | 190 | | | | |
| males | | 52 | 70.3 | 0.74 | 0.55–0.97 |
| females | | 50 | 68.3 | 0.73 | 0.54–0.97 |
| Other skin | 191 | | | | |
| males | | 53 | 61.1 | 0.87 | 0.65–1.13 |
| females | | 41 | 39.6 | 1.04 | 0.74–1.41 |
| Brain, nervous system | 193 | | | | |
| males | | 52 | 55.7 | 0.93 | 0.70–1.22 |
| females | | 34 | 48.7 | 0.70 | 0.48–0.98 |
| Thyroid gland | 194 | | | | |
| males | | 5 | 10.3 | 0.49 | 0.16–1.13 |
| females | | 19 | 25.4 | 0.75 | 0.45–1.17 |
| Non-solid tumours (200–204) | | | | | |
| males | | 134 | 144.2 | 0.93 | 0.77–1.09 |
| females | | 83 | 102.9 | 0.81 | 0.64–1.00 |
| All sites | | | | | |
| males | | 1786 | 1887.5 | 0.95 | 0.90–0.99 |
| females | | 1507 | 1678.4 | 0.90 | 0.85–0.94 |

diagnosed and regular matching with the mortality register (Statistics Norway), is of great importance in the completeness of the register. Thus, cancer registration effectively covers the total population.

The personal identity number was used to link the cancer registry with the twin birth cohort of the period 1905–1945. Information regarding emigration and death was also collected. For the analysis of cancer, each person was followed from 1 January 1960 until date of death, emigration, diagnosis of first malignant tumour or the end of the follow-up on 31st December 1996, whichever came first. Emigration represented less than 0.1%, with no other loss to follow-up. Our analyses are based on a

 *© 2001 Cancer Research Campaign*

comparison of the observed number of new cases of cancers in the cohort during the follow-up period with the expected numbers of the total population of the country and presented as the standardised incidence ratio (SIR = observed/expected) (Silva dos Santos, 1999).

### RESULTS

The total person-years of follow-up were 709 009. During the follow-up period from 1960 to 1996, a total of 3293 cases of cancer were observed, compared with an expected number of 3566, a reduced risk of malignant disease for all sites combined (Table 1). The SIR is 0.92 (95% confidence intervals 0.89–0.96).

With regard to the specific cancer sites, we observed a significant reduction of colon cancer in male twins and of rectal cancer in female twins, and a similar tendency in the opposite gender. In both sexes, there was a reduced incidence of malignant melanomas of the skin and non-solid tumours (leukaemia, lymphoma and myeloma). In females, central nervous system and lung tumours were reduced with a similar tendency among males and for cancer of the kidney in both sexes. No cancer showed a significant increased incidence except cancer of the ovary.

### DISCUSSION

For some types of cancer, being a twin seems to protect against malignant disease. A study from Finland covering the period 1976 to 1995 found a slight decrease in the total cancer incidence among twins (SIR 0.95, 95% CI 0.91–1.00) (Verkasalo et al, 1999). We have considered the possibility that our observations might be due to deficient sampling, but we find it unlikely that the reporting practices for cancer in twins should differ from those in non-twins.

The reduced incidence of cancer among twins might be the result of certain shared factors in childhood, such as dietary habits or other kinds of exposure and not genetic factors. Thus, data from 44 788 pairs of twins from Sweden, Denmark and Finland showed that inherent genetic factors made a minor contribution to most types of neoplasm (Lichtenstein et al, 2000), though in testis cancer a large genetic component together with perhaps nutritional factors were suggested as causes (Swerdlow et al, 1999).

There is growing evidence that some types of cancer may originate in utero; especially in hormone-related cancers (Tibblin et al, 1995; Akre et al, 1996; Ekbom et al, 1996, 1997, 2000; Michels et al, 1996; Ekbom 1998; Cerhan et al, 2000). The reported increased incidence of prostate cancer in the highest quartile of birth weight (Tibblin et al, 1995) was not confirmed in another study (Platz et al, 1998).

Our data include twins born during 1905–1945 who were alive in 1960. We have, however, lost information on cancers in children so we cannot confirm an earlier report showing an increased number of childhood kidney cancers in twins (Windham et al, 1985). We could not investigate the role of zygosity but we hope to do this later as have others (Swerdlow et al, 1997).

We have observed a reduced risk of cancer in twins, and we consider this a real finding which cannot be explained by deficient sampling. Twins are often born at an earlier gestation age than singletons and have a reduced birth weight. In Norway 1919–1930 the mean gestation for twins was 16 days shorter than in singletons, with a correspondingly lower birth weight (Waaler, 1934). We might therefore have found the same decreased incidence of malignant disease in singletons born at a similar early gestation age as the twins.

### REFERENCES

Akre O, Ekbom A, Hsieh CC, Trichopoulos D and Adami HO (1996) Testicular nonseminoma and seminoma in relation to perinatal characteristics. *J Natl Cancer Inst* **88**: 883–889

The Cancer Registry of Norway (1982) Evaluation of completeness of reporting. In: *Annual Report of the Cancer Registry* 19–20

Cerhan JR, Kushi LH, Olson JE, Rich SS, Zheng W, Folsom AR and Sellers TA (2000) Twinship and risk of postmenopausal breast cancer. *J Natl Cancer Inst* **92**: 261–265

Ekbom A (1998) Growing evidence that several human cancers may originate in utero. *Semin Cancer Biol* **8**: 237–244

Ekbom A, Hsieh CC, Lipworth L, Wolk A, Ponten J, Adami HO and Trichopoulos D (1996) Perinatal Characteristics in relation to incidence of and mortality from prostate cancer. *BMJ* **313**: 337–341

Ekbom A, Hsieh CC, Lipworth L, Adami HO and Trichopoulos D (1997) Intrauterine environment and breast cancer risk in women: a population based study. *J Natl Cancer Inst* **89**: 71–76

Ekbom A, Erlandsson G, Hsieh CC, Trichopoulos D, Adami HO and Cnattingius S (2000) Risk of breast cancer in prematurely born women. *J Natl Cancer Inst* **92**: 840–841

Harvei S, Tretli S and Langmark F (1996) Quality of prostate cancer data in The Cancer Registry of Norway. *Europ J Cancer* **32A**: 104–110

Kringlen E (1978) Norwegian twin registers. In: *Twin Research: Biology and Epidemiol.* New York: Alan R. Liss, Inc., 185–187

Lichtenstein P, Holm NV, Verkasalo PK, Iliadou A, Kaprio J, Koskenvuo M, Pukkala E, Skytthe A and Hemminki K (2000) Environmental and heritable factors in the causation of cancer. Analyses of cohorts of twins from Sweden, Denmark and Finland. *The New England Journal of Medicine* **343**: 78–85

Lund E (1981) Pilot study for the evaluation of completeness of reporting to The Cancer Registry. In: *Incidence of cancer in Norway 1978 The Cancer Registry of Norway* 11–14

Michels KB, Trichopoulos D, Robins JM, Rosner BA, Manson JE, Hunter DJ, Colditz GA, Hankinson SE, Speizer FE, Willett WC (1996) Birthweight as a risk factor for breast cancer *Lancet* **348**: 1542–1546

Platz EA, Giovannucci E, Rimm EB, Curhan GC, Spiegelman D, Colditz GA and Willett WC (1998) Retrospective analysis of birth weight and prostate cancer in the health professionals follow-up study. *Am J Epidemiol* **147**: 1140–1144

Stavola BL, Hardy R, Kuh D, Silva IS, Wadsworth M and Swerdlow AJ (2000) Birthweight, childhood growth and risk of breast cancer in a British cohort. *Br J Cancer* **83**: 964–968

Silva dos Santos I (1999) *Cancer Epidemiology: Principles and methods.* Lyon: International Agency for Research on Cancer

Swerdlow AJ, Stavola de BL, Swanwick MA and Maconochie NES (1997) Risks of breast and testicular cancers in young adult twins in England and Wales: evidence on prenatal and genetic aetiology. *Lancet* **350**: 1723–1728

Swerdlow AJ, De Stavola BL, Swanwick MA, Mangtani P and Maconochie NE (1999) Risk factors for testicular cancer: a case-control study in twins *Br J Cancer* **80**: 1098–1102

Tibblin G, Eriksson M, Cnattingius S and Ekbom A (1995) High birth weight as a predictor of prostate cancer risk. *Epidemiology* **6**: 423–424

Trichopoulos D and Lipworth L (1995) Is cancer causation simpler than we thought, but more intractable? *Epidemiology* **6**: 429–433

Verkasalo PK, Kaprio J, Koskenvuo M and Pukkala E (1999) Genetic predisposition, environment and cancer incidence: a nation-wide twin study in Finland, 1976–1995 *Int J Cancer* **83**: 743–749

Waaler GHM (1934) Om nyfødte tvillingers størrelse. (Abstract in German.) *Norsk Magasin for Lægevidenskapen* October 1113–1141

Windham GC, Bjerkedal T and Langmark F (1985) A population-based study of cancer incidence in twins and in children with congenital malformations or low birth weight, Norway, 1967–1980 *Am J Epidemiol* **121**: 49–56

© 2001 Cancer Research Campaign 
